# Mesoporous Silica Nanoparticles for Dual-Mode Chemo-Sonodynamic Therapy by Low-Energy Ultrasound

**DOI:** 10.3390/ma11102041

**Published:** 2018-10-19

**Authors:** Jingjing Wang, Yajing Jiao, Yiran Shao

**Affiliations:** 1 Key Laboratory of Inorganic Coating Materials CAS, Shanghai Institute of Ceramics, Chinese Academy of Sciences, Shanghai 200050, China; 18616938063@163.com (J.W.); 18800263296@163.com (Y.J.); 2 University of Chinese Academy of Sciences, Beijing 100049, China

**Keywords:** low-energy ultrasound, mesoporous silica, folic acid, rapid release, chemo-sonodynamic therapy

## Abstract

Low-energy ultrasound (LEUS), exhibiting obvious advantages as a safe therapeutic strategy, would be promising for cancer therapy. We had synthesized a LEUS-responsive targeted drug delivery system based on functional mesoporous silica nanoparticle for cancer therapy. Paclitaxel (PTX) was loaded in mesoporous silica nanoparticles with a hydrophobic internal channel, and folic acid (FA) functionalized β-Cyclodextrin (β-CD) was capped on the surface of the nanoparticles (DESN), which acted as a cancer-targeting moiety and solubilizer. The existence of a hydrophobic internal channel in the DESN was beneficial to the storage of hydrophobic PTX, along with the enhancement of the cavitation effect produced by mild low-energy ultrasound (LEUS, ≤1.0 W/cm^2^, 1 MHz). The DESN showed significantly enhanced cavitation effect, selective targeting, and achieved a rapid drug release under mild LEUS. To investigate the in vivo antitumor efficacy of the DESN upon LEUS irradiation, we established a 4T1 mammary tumor model. The DESN were confirmed to be of great biodegradability/biocompatibility. The tumor growth was significantly inhibited when the mice were treated with DESN (10 mg/kg) + LEUS with the relative tumor volume reduced to 4.72 ± 0.70 compared with the control group (V/V_0_ = 17.12 ± 2.75). The DESN with LEUS represented excellent inhibiting effect on tumor cell in vivo. This work demonstrated that DESN mediating dual mode chemo-sonodynamic therapy could be triggered by extracorporeal remote control, may suggest a promising clinical application in cancer therapy.

## 1. Introduction

Cancer is now one of the most serious diseases threatening the health of human beings. Cooperative therapeutic strategies, which may overcome the intrinsic shortages of traditional chemotherapy, have drawn great attention in recent years [1,2,3]. For example, chemotherapy assisted by ultrasound based on mesoporous silica nanomaterials has been intensively investigated for improving cancer treatment [4,5].

Ultrasound recently aroused increasing interest as a preferable tool for cancer diagnostics because of its noninvasiveness, nonionizing nature, cost-effectiveness and easy regulation of tissue penetration depth by tuning the frequency, cycles, and exposure time [6,7]. Additionally, ultrasound was one of the most promising external triggers for achieving spatiotemporal control of the drug release at the desired site, and has been shown to enhance nanoparticles’ extravasation through blood capillaries, increase cell membrane permeation, and even induce an immune response against tumors [6,8,9]. Aside from these features, ultrasound waves can damage or even kill tumor cells by inducing thermal effect, sonochemistry, and mechanical effect [10,11]. In fact, high-intensity focused ultrasound (HIFU) had demonstrated an ability to produce tissue necrosis by converting ultrasound energy into therapy and mechanical energy [12,13,14,15]. Low-energy ultrasound has attracted great attention in the cancer treatment due to being more secure and easier to operate compared to HIFU [16,17].

Previous research demonstrated that the responses of malignant cells to LEUS (with typical intensities of 0.1–2 W/cm^2^, and frequencies of 0.5–3 MHz) are more sensitive than those of normal tissue cells [18,19], and LEUS technology was initially used in medicine for physiotherapy applications [20,21,22]. Nevertheless, the energy of low-energy ultrasound is too low to overcome the tensile strength of water to produce a new cavity in the absence of cavitation micronuclei [23,24]. In order to enhance the cavitation effect, a series of ultrasonic sensitizer (organic or lipid microbubbles) was successfully designed and fabricated recently [25,26,27]. Compared to microbubbles, the mesoporous silica (MSN) framework possessed high structural stability in the body circulation, which ensured highly efficient drug delivery into the tumor. The structure with mesoporous channels had been confirmed to allow controllable drug release by ultrasonic irradiation through a mechanical acceleration effect [5,28], and enhanced the cavitation effect [29,30]. Dual-effect nanoparticles, with the function of accelerating drug release and enhancing cavitation effect under LEUS, could be designed using mesoporous silica. 

In this study, we synthesized PTX-loaded and FA-β-Cyclodextrin (FA-β-CD)-capped mesoporous silica nanoparticles with a hydrophobic internal channel. A folic acid (FA) molecule, which has been known as a targeting reagent for cancer cells, was functionalized on the β-CD by the mediation of APTES. MSN with abundant mesoporous channels were utilized as carriers to load hydrophobic PTX and store gas in the hydrophobic internal channel as an ultrasonic cavitation nucleus. The final product PTX@FA-β-CD/H-MSN (DESN) enhanced the cavitation effect and achieved rapid drug release under mild LEUS. The biocompatibility and chemo-sonodynamic efficiency of DESN upon LEUS were systematically evaluated in vitro/vivo. Our results indicated that DESN combined with LEUS irradiation had a significant killing performance at a cellular level and excellent inhibiting effect at the animal level. The DESN can serve as an excellent nanoplatform for dual-mode chemo-sonodynamic therapy in cancer therapy [31].

## 2. Experimental Section

### 2.1. Materials

Cetyltrimethyl ammonium bromide (CTAB), Paclitaxel (PTX), Hexamethyl disilylamine (HMDS), beta-cyclodextrin (β-CD), tetraethyl orthosilicate (TEOS), terephthalic acid (TA), folic acid (FA), (3-aminopropyl) triethoxysilane (APTES), 1-(3-Dimethylaminopropyl)-3-ethylcarbodiimide hydrochloride (EDC), Sodium hydroxide (NaOH), N-hydroxysuccinimide (NHS), and fluorescein isothiocyanate (FITC) were obtained from Aladdin Chemistry Co., Ltd. (Shanghai, China). MTT was purchased from Sigma–Aldrich (St. Louis, MO, USA). 4′,6-Diamidino-2-phenylindole (DAPI) was purchased from Nanjing Key Gen Biotech Co., Ltd. (Nanjing, China).

### 2.2. Synthesis of Dual-Effect Mesoporous Silica Nanoparticles (DESN)

In a typical procedure, mesoporous silica nanoparticles (MSN) were prepared according to previous studies [32,33]. Briefly, 1.0 g CTAB was dissolved in a solution of 480 mL deionized water and NaOH (2 M, 3.5 mL), stirred for 2 h at 80 °C. After that TEOS (5 mL) was added dropwise, and the mixture was vigorously stirred for 6 h at 80 °C. The white products were collected by centrifugation and washed with ethanol and deionized water several times. Finally, the products were suspended in 100 mL of ethanol containing 10 mL of HCl (1 M) to remove the template CTAB. After refluxing at 60 °C for 8 h, the MSN were obtained by centrifugation and washed with deionized water and ethanol. This procedure was repeated several times until the templates were removed completely. Hydrophobic MSN with hydrophobic internal channel (H-MSN) were prepared by adding HMDS (1.3 mL) to a suspension of MSN (4 mg/mL, 25 mL) in n-hexane. After stirring for 24 h at 25 °C, the resulting products H-MSN were obtained by centrifugation, washing and drying [32]. Then, PTX was incorporated into H-MSN through free diffusion. H-MSN was added into the DMSO solution containing PTX (1 mg·mL^−1^), and the mixture solution was stirred for 24 h in the dark. After centrifugation and washing with DMSO and deionized water, PTX@H-MSN was dried in a vacuum. 

FA-β-CD was prepared for further use. Firstly, β-CD (500 mg) were dispersed in 40 mL of toluene, and the solution was refluxed for 8 h, then 500 μL APTES was added in. The mixture solution was stirred at 50 °C for 8 h. After centrifugation and washing with ethanol, β-CD-NH_2_ was dried in vacuum. Secondly, FA (130 mg), DEC (68 mg) and NHS (76 mg) were dispersed in 30 mL of DMSO, and the solution was stirred at 50 °C for 6 h. Finally, β-CD-NH_2_ (500 mg) was added into the above solution, and the reaction lasted for another 24 h at the same temperature. After centrifugation and washing with DMSO and deionized water, the resulting products FA-β-CD was dried at 40 °C in vacuum.

To obtain DESN, PTX@H-MSN (100 mg) was added to the solution of FA-β-CD (0.3 g) in deionized water (50 mL). After vigorous stirring for 12 h at 25 °C, FA-β-CD (0.2 g) was added to the above solution and stirring continued for another 12 h. DESN were collected by centrifugation, followed by washing with H_2_O and freeze-drying.

### 2.3. Evaluation of PTX Releasing Profiles under LEUS with Different Intensity

The PTX release profiles were monitored by UV/Vis spectroscopy (TECHCOMP, Hong Kong, China). DESN with accurate weight were immersed in four same PBS solutions (pH = 7.4) and treated under low-energy ultrasound irradiation with different intensities (1 MHz; 0.4, 0.6, 0.8, and 1.0 W·cm^−2^) for 2 min. After a given time interval, the supernatant (1 mL) was extracted from each beaker and replaced with a fresh release medium (1 mL). The PTX release profile of MSN and DESN with untreated LEUS was evaluated by the same method.

### 2.4. Assay of Intensity of Ultrasound Cavitation by Fluorescence Spectrometry

The TA dosimeter (5 mM TA, 10 mM NaOH) was routinely prepared in PBS (pH = 7.4) as a substrate solution and stored in brown bottles at 4 °C. Prior to use, the solution should be warmed to room temperature. MSN and DESN were dispersed in the TA-PBS solution (20 mL, 1 μg·mL^−1^), respectively. Then, the solutions were exposed to ultrasound irradiation for 3 min with different intensities (0.4, 0.6, 0.8, and 1.0 W·cm^−2^). The fluorescence spectrometry of the solutions was obtained under excitation with UV light (310 nm).

### 2.5. Cell Culture

The mouse breast cancer line 4T1 cells were purchased from the cell bank of the Chinese Academy of Science. Cells were cultured in RPMI-1640 medium (pH = 7.4), supplemented with 10% fetal bovine serum (FBS) and 1% penicillin streptomycin combination. All cells were maintained in a humidified incubator under 5% CO_2_ at 37 °C.

### 2.6. Cellular Uptake of Fluorescently Labeled DESN

DESN were labeled with fluorescein isothiocyanate FITC to visualize the uptake of the nanosystem by cancer cells. FITC-APTES was prepared by the addition reaction between FITC (10 mg) and APTES (24 μL) in methanol (4 mL) for 24 h under light-sealed and dry conditions at room temperature. Then, FITC-DESN were obtained through the successive addition of FITC-APTES (2 mL) and DESN (20 mg). 4T1 cells (6 × 10^4^ cells per dish) were seeded in Petri dishes and cultured for 12 h at 37 °C. After incubation with FITC-DESN (25 μg·mL^−1^) for 12 h, the medium was discarded and the cells were washed twice with PBS to remove residual nanoparticles. Subsequently, 0.5 mL of DAPI in methanol (10%) was added and the cells were incubated for 15 min to stain the nuclei and fix the cells. Then the cells were gently washed twice with methanol to remove excessive DAPI. Finally, the cells were visualized by fluorescence microscopy (Olympus IX71, Olympus, Tokyo, Japan).

### 2.7. In Vitro Cytotoxicity Assay

MTT reduction assay was performed to evaluate the in vitro cytotoxicity of DESN against 4T1 mouse breast cancer cells. The 4T1 cells were seeded in 96-well plates at a density of 5.0 × 10^4^ cells·mL^−1^ per mL and were cultured in the RPMI-1640 medium supplemented with 10% FBS, 1% penicillin streptomycin, and samples of DESN with the concentration of 0, 5, 10, 20, and 40 μg·mL^−1^. After co-culturing for another 12 h, the mixture was discarded and the cells were washed twice with PBS. Then 20 μL of MTT solution was added in each well and the cell viability was measured by Thermo fisher microplate reader at 490 nm. Similar experiments were performed on 4T1 cells to further evaluate the cytotoxicity of MSN and FA-β-CD/H-MSN.

To investigate the cytotoxicity of nanoparticles under LEUS, the cells were treated with DESN and exposed to LEUS. Firstly, the as-prepared cells were cultured with DESN with the concentration of 5, 10, 20 and 40 μg·mL^−1^. After a 12 h co-culture, the 96-well plates were treated with LEUS at different ultrasound intensities (from 0.4 W·cm^−2^ to 1.0 W·cm^−2^) for 80 s. After that the cells were cultured for another 12 h and the cell viability was determined by the MTT assy. Data are represented as mean ± standard deviation (SD) of six independent experiments (*n* = 6, *n* indicates the number of wells in the plate for each experimental condition).

### 2.8. In Vivo Cytotoxicity Test

Kunming mice were obtained from the Changhai hospital and were housed ender specific pathogen-free conditions. All experimental procedures and euthanasia were done painlessly in compliance with the Guide for the Care and Use of Laboratory Animals Healthy female Kunming mice (~30 g) were housed in an isolated biosafety facility for specific pathogen-free conditions for 7 days, and were divided into four groups (*n* = 3). The mice were injected with DESN at different doses (0, 5, 10, 20 mg·kg^−1^). The body weights of mice were measured every 2 days for a one-month period. After that, the mice were sacrificed and their blood samples and organs (heart, liver, spleen, lung, and kidney) were taken out for different tests regarding biocompatibility.

### 2.9. In Vivo Antitumor Effect of DESN upon LEUS Irradiation

Female BALB/c nude mice at five weeks of age were obtained from the Shanghai Laboratory Animal Center and were housed in an isolated biosafety facility for specific pathogen-free conditions. All experimental procedures and euthanasia were carried out in accordance with the Guide for the Care and Use of Laboratory Animals.

Under sterile conditions, BALB/c nude mice were injected with 1 × 10^6^ cells·mL^−1^ in vitro propagated 4T1cells subcutaneously in the lower flank of mice. Experiments were started when the tumors volume reached around 100 mm^3^. Firstly, the tumor-hearing BALB/c nude mice were divided into three groups (*n* = 5), three groups of mice were treated with PBS, DESN (5 mg/kg) + LEUS and DESN (10 mg/kg) + LEUS, respectively. DESN dissolved in 0.5 mL PBS was injected into tumors at a dose of 10 μL every two days. The DESN + LEUS groups were applied a low-energy ultrasound irradiation (0.8 W·cm^−2^, 1 MHz) through a water bag (ca. 20 mm) for 3 min plus 2 min with a pause of 2 min to prevent any increase in temperature (High water temperature affects the results, so the time setting is based on not causing the temperature of water bag to rise). The tumor volume and body weight were monitored every other day over 15 days. At the end of treatment, the tumor was dissected, the tumors and major organs were collected and stained by hematoxylin and eosin (H&E), TdT-mediated dUTP Nick-End Labeling (TUNEL), and Ki-67 for histological analysis. The tumor volume (V) was calculated as follows:

(1) $$\;V=\frac{L\times W^{2}}{2},$$

where L and W refer to the longest dimension and the shortest dimension of tumor, respectively.

The therapeutic effect was assessed using the individual relative tumor volume (RTV), which was calculated as follows:

(2) $$\mathrm{RTV}=\frac{V}{V_{0}},$$

where V is the measured volume every two days, and V_0_ is the initial volume at the beginning of the treatment.

### 2.10. Characterization

The morphology and size of the samples (MSN and DESN) were obtained by transmission electron microscopy (TEM, JEM-2010, JEOL, Tokyo, Japan) with an accelerating voltage of 200 kV. A powder X-ray diffraction (XRD) pattern was recorded on a diffractometer (Ultima IV, Rigaku, Wilmington, MA, USA) with Cu Ka radiation. Fourier transform infrared spectroscopy (FTIR, Shimadzu, Kyoto, Japan) was carried out using KBr discs in the region of 4000–400 cm^−1^. N_2_ adsorption–desorption isotherm measurement was carried out on a Micromeritics Tristar 3000 nitrogen adsorption apparatus. Surface area and pore volume were determined by BET analysis. Thermogravimetric (TG, NETZSCH STA 449 c, NETZSCH, Paris, France) were carried out on a TA thermal analyzer at a heating rate of 5 °C·min^−1^ in air. The UV/Vis adsorption spectrum was measured by a UV-2300 spectrophotometer (TECHCOMP, Hong Kong, China). Photoluminescence (PL) spectra were recorded on a FluoroMax-4 fluorescence spectrometer. Cell morphology was analyzed using a fluorescence microscope (Olympus IX71, Olympus, Tokyo, Japan). The zeta potential of samples were measured by Malvern Zetasizer Nano ZS model ZEN3690 (Malvern, Worcestershire, UK).

## 3. Result and Discussion

### 3.1. Preparation and Characterization of the DESN Drug Delivery System

**Scheme 1:**
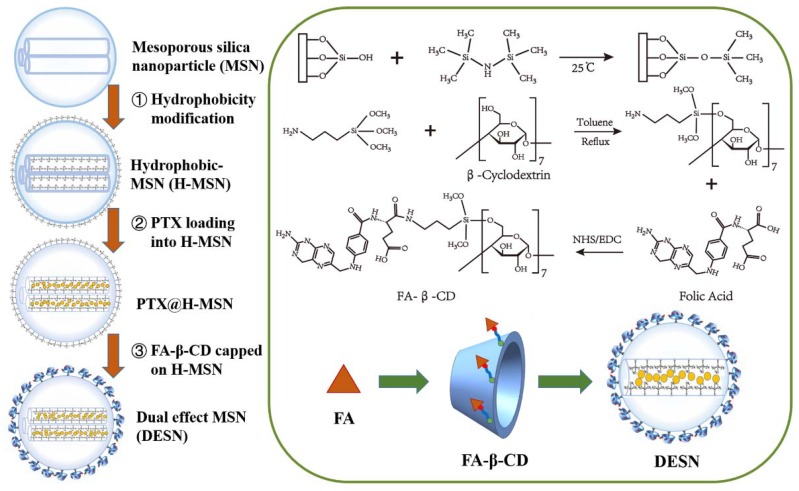
Schematic illustration of the synthesis of DESN. The hydrophobic SiMe_3_ groups were grafted onto the surface of MSN through an alkylation reaction, and PTX was incorporated into H-MSN through free diffusion. Finally, the hydrophobic SiMe_3_ groups on the surface of the PTX@H-MSN were partially capped by FA-β-CD.

The whole procedure of the DESN drug delivery system was illustrated in Scheme 1. In this study, mono-dispersed MSN were fabricated based on a previous study [29]. Then, the hydrophobic SiMe_3_ groups were grafted on the surface of MSN through an alkylation reaction for storing more gas. β-cyclodetrin (β-CD) was conjugated with folic acid molecules through APTES, with further coating on the outer surface of MSN by hydrophobic adsorption. Finally, PTX was loaded into the channel of FA-β-CD/H-MSN, a process that created what are named double-effect silica nanoparticles (DESN). It can be seen from Scheme 1 that β-CD acted as the cap of the channel.

**Figure 1:**
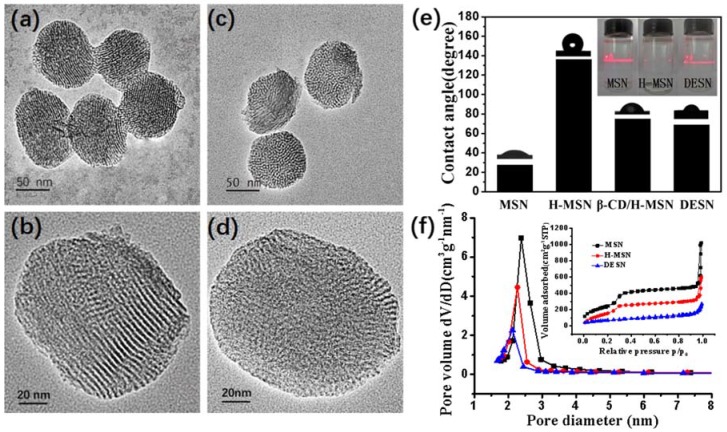
TEM images of MSN: (**a**,**b**) FA-β-CD/H-MSN; (**c**,**d**) Contact angle between nanoparticles (MSN, H-MSN, β-CD/H-MSN and DESN) and water in air, and the Tyndall effect of nanoparticles (MSN, H-MSN, and FA-β-CD/H-MSN) dispersed in water (the inset); (**e**) The pore size distribution curve and N_2_ sorption isotherms (the inset) of MSN, H-MSN, and DESN and (**f**) The N_2_ adsorption‒desorption measurement was adopted to expose the effect of the β-CD coating and PTX storage on the porosity of MSN.

The morphology of MSN before and after surface modification was evaluated by TEM. As shown in Figure 1a, the as-prepared MSN were well-dispersed, with an average diameter of about 120 nm. The magnified TEM image in Figure 1b indicated that the spheres had an ordered mesoporous channel with an aperture of 2–3 nm. To increase the water solubility of nanoparticles and control release of PTX, FA-β-CD was introduced onto the MSN. The ordered mesopore structure of the DESN could not be clearly observed (Figure 1c,d), which indicated the successful coating of FA-β-CD on the MSN.

Previous studies suggested that when two contiguous hydrophobic surfaces are separated, they are connected through a gas bridge. The interfacial free energy of a hydrophobic surface is lower against gas than against water, so it is energetically favorable to replace water with gas between the hydrophobic surface [34]. A bright “path” appeared in the solution of both MSN and DESN under laser irradiation, while it was not observed in the solution of H-MSN. The detection of the Tyndall effect also proved that DESN can be well dispersed in the solution (Figure 1e, inset). The contact angle between H-MSN and water in air reached about 135°. The MSN was re-dispersible in water when being externally capped by FA-β-CD (Figure 1e). β-CD has a hydrophobic cavity, which makes it feasible to cap the hydrophobic SiMe_3_ groups present on the external surface of MSN (Scheme 1).

N_2_ adsorption‒desorption measurement was adopted to reveal the effects of the β-CD coating and PTX storage on the porosity of MSN. As shown in Figure 1f and Table 1, these samples possessed a typical type IV isotherm with a type H2 hysteresis loop, which demonstrated the presence of a well-defined mesoporous structure. Thus, almost no relative pressure (P/P_0_) leap between 0.2 and 0.35 was observed in DESN, further demonstrating PTX loading and FA-β-CD coating. The Brunauer-Emmett-Teller (BET) surface area and Barrett-Joyner-Halanda (BJH) pore volume of MSN were calculated to be 1000 m^2^·g^−1^ and 1.089 cm^3^·g^−1^, respectively, which were significantly higher than those of the DESN (247m^2^·g^−1^ and 0.28 cm^3^·g^−1^). The corresponding pore size was reduced from 2.38 nm to 2.17 nm (Table 1). Zeta potential analysis was used to detect the potential changes before and after surface modification. As shown in Table 1, with the different modifications of MSN, zeta potential changes. All the results indicated that the surface was successfully modified.

Combined with the low-angle XRD diagram in Figure S1, both patterns showed three well-resolved Bragg diffraction peaks, which can be assigned to the (100), (110), and (200) reflections of a hexagonal symmetry structure (P6mm) typical. These results indicated that all the sample have an ordered pore structure, while the intensity of the diffraction peaks decreased as PTX was loaded. The results showed that FA-β-CD had been successfully grafted on the surface, and the drug was successfully stored in the modified nanoparticles.

**Figure 2:**
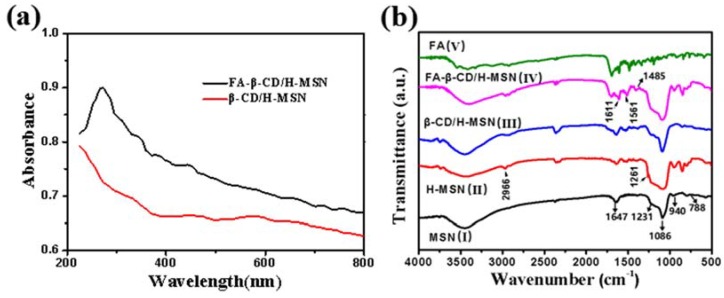
(**a**) Absorption spectra of β-CD/H-MSN (red) and FA-β-CD/H-MSN (black). Comparing with the spectrum of β-CD/H-MSN, the spectrum of FA-β-CD/H-MSN had a special peak at 272 nm, indicating the successful modification of folic acid; (**b**) FT-IR spectra of MSN, H-MSN, β-CD/H-MSN, FA-β-CD-H-MSN, and FA (bottom to top). The emergence of the bands at 1611 and 1516 cm^−1^ is due to amide I and amide II bonds, which further illustrated the successful connection of FA.

In addition, Figure 2a shows the UV absorption spectra of β-CD/H-MSN and FA-β-CD/H-MSN. Comparing with the spectrum of β-CD/H-MSN, the spectrum of FA-β-CD/H-MSN had a special peak at 272 nm, which was attributed to the π‒π* transition of the pterin ring of the molecule [35]. To further detect the presence of FA in FA-β-CD/H-MSN, FT-IR spectroscopy was used to monitor the surface modification of MSN. Figure 2b showed the FTIR of samples MSN(I), H-MSN(II), β-CD/H-MSN (III), FA-β-CD/H-MSN(IV), and FA(V). As shown in Figure 2b (I), the MSN displayed characteristic bands of silica at 1647, 1231, 1086, 940, and 788 cm^−1^, which can be attributed to the asymmetric stretching vibrations of Si‒O‒Si at 1231 and 1086 cm^−1^. The symmetric stretching vibration of Si‒O‒Si was at 788 cm^−1^, the stretching vibration of Si‒OH was at 940 cm^−1^, and the peak at 1647 cm^−1^ was attributed to physically absorbed H_2_O in MSN. To test the successful connection of folic acid, comparing the FT-IR spectrum of FA with those of FA-β-CD/H-MSN, both had intensity absorption sited at 1695, 1607, and 1485 cm^−1^. The emergence of the bands at 1611 cm^−1^ and 1561 cm^−1^ was due to amide I and amide II bonds [36,37]. The peaks between 1485 cm^−1^ and 1513 cm^−1^ were assigned to characteristic absorption and of the PT ring and phenyl. The above data indicated that FA was successfully conjugated in FA-β-CD/H-MSN.

### 3.2. Evaluation of the Enhancement of the Cavitation Effect

**Figure 3:**
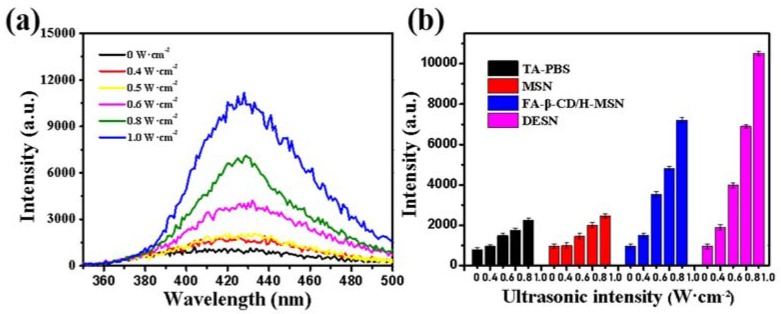
(**a**) Fluorescence assay of the cavitation effect in dispersions of DESN in PBS after ultrasound irradiation for 2 min (1 MHz; 0, 0.4, 0.6, 0.8, and 1.0 W·cm^−2^); (**b**) Fluorescence assay of the cavitation effect in dispersions of no nanoagents, MSN, FA-β-CD/H-MSN, and DESN in PBS under different ultrasound irradiation intensities ((1 MHz; 0, 0.4, 0.6, 0.8, and 1.0 W·cm^−2^) for 2 min. The results depicted were represented as means ± SDs (*n* = 3).

The phenomenon of ultrasonic cavitation under LEUS was detected using fluorescence spectroscopy (Figure 3). Ultrasonic cavitation consequently creates **·**OH radicals and H atoms via dissociation of water, and non-fluorescent terephthalic acid (TA) becomes highly fluorescent hydroxyl terephthalic acid (PTA) when combining with a hydroxyl radical **·**OH [38,39]. The intensity of the cavitation effect was positively correlated with the fluorescence intensity. The fluorescence intensity of a TA-PBS solution containing DESN was about 4 times higher than that of TA-PBS solution with or without MSN when exposed to ultrasound (1 MHz) with an intensity of 0.8 W·cm^−2^ for two minutes (Figure 3). The fluorescence intensity increased significantly when the ultrasonic intensity reached 0.6 W·cm^−2^, indicating that the cavitation threshold is 0.6 W·cm^−2^. However, due to the low energy, the addition of MSN fails to reach the cavitation threshold, resulting in a change in the cavitation effect. Moreover, the fluorescence intensity of the PT-PBS solution containing DESN was further improved compared with that of FA-β-CD/H-MSN. The cause of this phenomenon could be attributed to three points. Firstly, DESN with internal hydrophobic channels ensures the entrapment and stability of gas bubbles as nucleation seeds. Furthermore, the hydrophobic interface could also reduce the tensile strength of water [29,40]. The last point was due to the loading of hydrophobic drug paclitaxel.

### 3.3. Evaluation of LEUS-Responsive PTX Release Profiles under LEUS with Different Intensities (1 MHz; 0, 0.4, 0.6, 0.8, and 1.0 W·cm^−2^)

The pharmacological activity of PTX-loaded nanoparticles (PTX@MSN and DESN) were evaluated by the PTX release assay in an PBS solution. Such PBS was chosen to mimic the environment of normal tissue/blood (pH = 7.4) [41]. The loading capacity of PTX monitored by UV-Vis absorption measurement was 12.4% in MSN and 14.83% in DESN (Table 1). TG curves of FA-β-CD/H-MSN and DESN were illustrated in Figure S2. By the comparison of curve c with curve d, it could be concluded that the weight loss in curve d was about 15.98%, owing to the volatilization and decomposition of PTX. The loading content of PTX was basically consistent with the results in Table 1.

**Figure 4:**
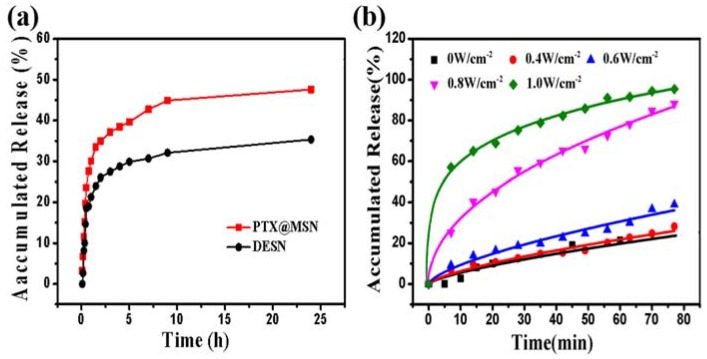
(**a**) The release profiles of PTX from MSN and DESN in PBS at pH 7.4; (**b**) the release profiles of PTX from DESN under LEUS with different intensities (1 MHz; 0, 0.4, 0.6, 0.8, and 1.0 W·cm^−^^2^). The existence of β-CD had a certain effect on PTX encapsulation, and the release rate of PTX was getting faster with the increasing of ultrasonic intensity. PTX cumulative release amount from DESN was about 21.48%, 31.01%, 76.76%, and 90.38% under ultrasonic intensities 0.4 W·cm^−2^, 0.6 W·cm^−2^, 0.8 W·cm^−2^, and 1.0 W·cm^−2^ after 60 min, respectively.

As is shown in Figure 4a, the release of PTX from MSN and DESN exhibited a typical sustained manner, whereas the release rate was distinctly different. The release rate of PTX from MSN was faster than that from DESN in PBS at pH 7.4. The cumulative release amount was about 35.3% for PTX@MSN and 47.6% for DESN after 24 h. The uncoated pore of MSN had no confinement of PTX from the mesoporous channels. By contrast, after coating the FA-β-CD layers, the PTX release form was distinctly different.

Moreover, Figure 4b presented the PTX release profiles from DESN under different intensities of LEUS (1 MHz; 0, 0.4, 0.6, 0.8, and 1.0 W·cm^−2^). It was clearly observed that the PTX release was confined and analogous under LEUS with different intensities. It took about 1 h when the release rate was reached about 18% at untreated, but the release amount was about 21.48%, 31.01%, 76.76%, 90.38% under ultrasonic intensities 0.4 W·cm^−2^, 0.6 W·cm^−2^, 0.8 W·cm^−2^, 1.0 W·cm^−2^ after 60 min, respectively. It was attributed that β-CD could partially block the pores of MSN and hinder the release of PTX, further to increase the stability of PTX. The PTX molecule released from the DESN, when FA-β-CD was degenerated from the surface of DESN under continuous LEUS. Meanwhile, acoustic cavitation introduced intense mechanical strain, increasing release substantially [42,43]. It could be concluded that DESN could deliver more PTX into tumor cells and reduce the loss of PTX during the process of transportation under LEUS.

### 3.4. In Vitro Antitumor Efficacy

**Figure 5:**
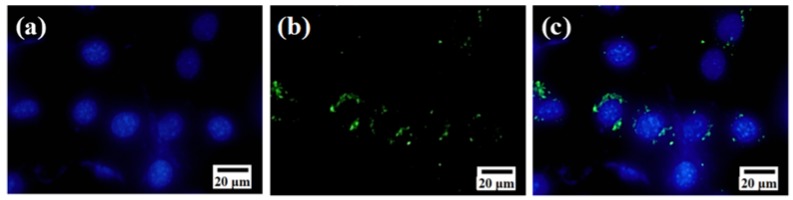
In vitro cellular uptake of DESN (25 μg·mL^−1^) in 4T1 cells: (**a**) Blue fluorescence image of cell nuclei stained with DAPI; (**b**) Green fluorescence image of FITC-labeled β-CD/H-MSN in cells and (**c**) Merged fluorescence image of (**a**,**b**).

To examine the cell-uptake property of DESN, nanoparticles were grafted with fluorescein isothiocyanate (FITC) for fluorescence labeling. After treating with FITC-DESN, 4T1 cells were fixed and the nuclei were stained with DAPI. The distribution of FITC-DESN in the cancer cells was observed by fluorescence microscopy (Figure 5). After incubation for 12 h, green fluorescence was distributed on and around the blue fluorescent nuclei, which indicated the DESN were mainly located in the cytoplasm and the perinuclear region. The results demonstrated a highly efficient uptake of DESN by 4T1 cells.

**Figure 6:**
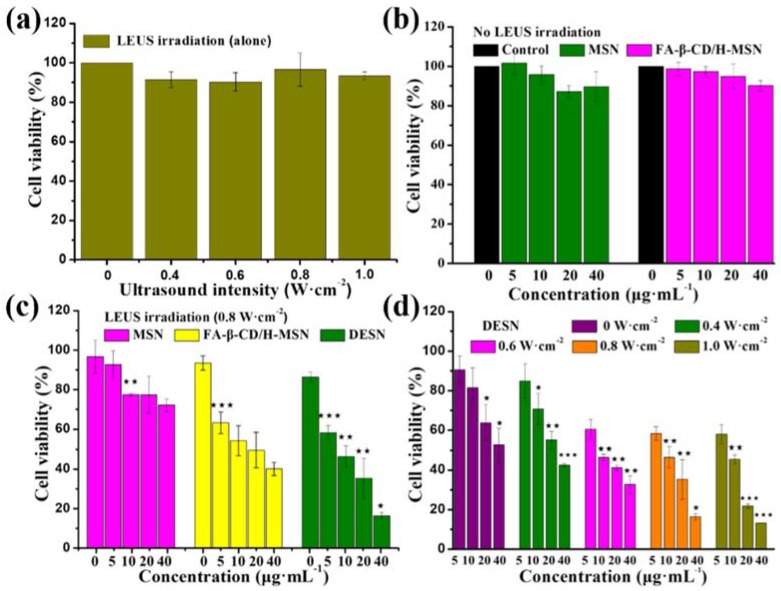
(**a**) Viability of 4T1 cells under low-energy ultrasound alone (1 MHz; 0, 0.4, 0.6, 0.8, and 1.0 W·cm^−2^); (**b**) in vitro cytotoxicity of MSN and FA-β-CD/H-MSN against 4T1 cells after a 24-h co-culture. The concentration of MSN was equal to FA-β-CD/H-MSN. Compared with the control group, MSN and FA-β-CD/H-MSN exhibited almost no cytotoxicity; (**c**) The viability of 4T1 cells after being co-cultured with MSN, β-CD/H-MSN, and DESN at different concentration (0, 5, 10, 20, and 40 μg·mL^−1^) under 0.8 W·cm^−2^ ultrasound irradiation and (**d**) Viability of 4T1 cells after co-culture with DESN with or without LEUS irradiation. FA-β-CD/H-MSN in synergistic combination with low-energy ultrasound (≤1.0 W·cm^−2^, 1 MHz) exerted enhanced toxicologic effects against tumor cells. DESN combined low-energy ultrasound with chemical therapy, making obvious the cell-killing effect. The results depicted were represented as means ± SDs (*n* = 3). ^★^ *p* < 0.05; ^★★^ *p* < 0.01;^★★★^ *p* < 0.001.

The cytotoxicity effects of MSN, FA-β-CD/H-MSN, DESN in vitro against 4T1 cells were investigated by MTT assays. Culture media without samples were used as the control. Little difference could be seen among the untreated or ultrasonic radiation of different intensities (1 MHz; 0, 0.4, 0.6, 0.8, and 1.0 W·cm^−2^) alone (Figure 6a), indicating that LEUS exhibited low cytotoxicity against 4T1. As shown in Figure 6b, without ultrasound irradiation, both MSN and FA-β-CD/H-MSN exhibited no obviously cytotoxicity against 4T1 cells, even at concentration of 40 μg·mL^−1^ after a 24-h co-culture. This indicated that MSN and FA-β-CD/H-MSN showed excellent biocompatibility [44,45]. Notably, the cell viability after a 24-h co-culture in RPMI-1640 media was 77% with MSN, and 50% with FA-β-CD/H-MSN at 20 μg·mL^−1^ plus 0.8 W·cm^−2^ ultrasonic radiation. Similar results were obtained at nanoparticle concentrations of 5, 10, and 40 μg·mL^−1^ under ultrasonic radiation of different intensity, which fully demonstrated that the addition of nanoparticles showed different cytotoxicity owing to the formation of the cavitation effect with different intensities.

Compared with the results of the pre-experiment, the DESN combined with low-ultrasound irradiation exhibited significantly high cytotoxicity (Figure 6d). The cell viability was 85% (0.4 W·cm^−2^), 60% (0.6 W·cm^−2^), 58% (0.8 W·cm^−2^), and 58% (1.0 W·cm^−2^) at 5 μg·mL^−1^ of DESN. With the concentration of DESN increased from 5 μg·mL^−1^ to 40 μg·mL^−1^, the cell viability decreased to 58%, 46%, 35%, and 16% upon 0.8 W·cm^−2^ LEUS irradiation, respectively. The intensity of LEUS also affected the cell viability except the concentration. With the irradiation dose increasing, the cell-killing effect increased.

**Figure 7:**
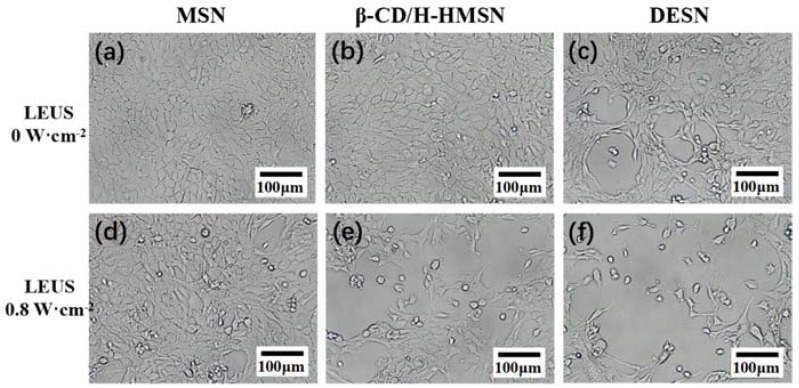
Microscopy images of 4T1 cells with no LEUS irradiation after co-culture with MSN (**a**); FA-β-CD/H-MSN (**b**) and DESN (**c**), respectively. Compared with the MSN group and FA-β-CD/H-MSN group, the morphology of the 4T1 cells in the DESN group slightly changed, which indicated low cytotoxicity. Microscopy images of 4T1 cells with LEUS irradiation (1 MHz, 0.8 W·cm^−2^) after co-culture with MSN (**d**); FA-β-CD/H-MSN (**e**) and DESN (**f**), respectively. Compared with those groups without LEUS, after nanoparticle incubation with DESN and exposure to LEUS irradiation (0.8 W·cm^−2^), the morphology of cells and population changed. However, after incubation with DESN and exposure to LEUS irradiation (0.8 W·cm^−2^), an obviously reduced cell population and changed cell morphology could be observed.

It could be seen in Figure 7 that an obviously reduced cell population and changed cell morphology could be observed after incubation with DESN and exposure to LEUS irradiation (0.8 W·cm^−2^). When the concentration of DESN was up to 40 μg·mL^−1^, the cell viability was reduced to 16%. Previous studies showed that the uptake of silica nanoparticles occurs through nonspecific adsorptive endocytosis or fluid-phase pinocytosis. Moreover, low-energy ultrasound itself could increase the permeability of the cell membrane [46,47]. The combination of LEUS with DESN showed a significant cavitation effect and produced obvious tumor cell-killing ability. This was attributed to the accelerated release of PTX from DESN with the assistance of LEUS. All the above results revealed that the combination of chemotherapy with ultrasonic therapy is more desirable for the effective treatment of cancer.

### 3.5. Safety Evaluation of DESN and In Vivo Tumor-Growth Inhibition Experiment

Potential in vivo toxicity is always a great concern when nanomaterials are used in biomedicine. In this work, an in vivo safety evaluation was carried out on Kunming mice at an elevated dose of DESN (0, 5, 10, and 20 mg/kg) by intravenous injection. The body weights of mice were recorded every 2 days for a one-month period. After that, mice were sacrificed to analyze the complete blood data while the organs (heart, liver, spleen, lungs, and kidneys) were dissected for histopathological examinations. Neither death nor significant body weight drop was noted in the DESN (Figure S3a). Furthermore, the corresponding histological changes in the major organs, including the heart, liver, spleen, lungs, and kidneys, were collected and sliced for hermatoxylin and eosin (H&E) staining (Figure S4). No significant acute, chronic pathological toxicity or adverse effects were found during the treatment period for all groups, suggesting no significant histological abnormalities in the treatment groups. The complete blood data, including blood routine examination (Figure S3b–f) and blood biochemistry indexes (Figure S3g–i), also indicated that no significant abnormality had been induced in venous blood after the intravenous injection of DESN. These preliminary data indicated the high biocompatibility of as-prepared DESN, and promised further explorations of using DESN for in vivo biomedical applications.

**Scheme 2:**
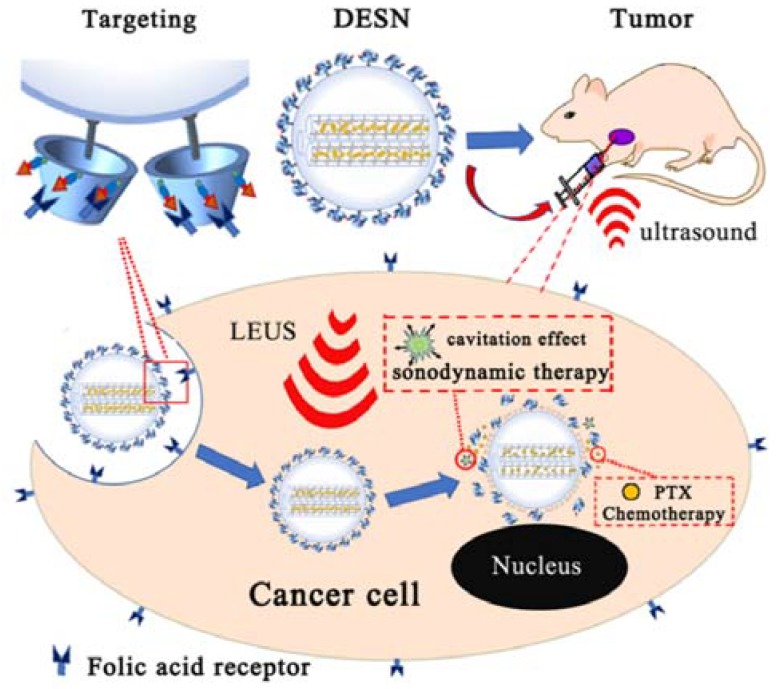
Schematic illustration of the process of killing tumor cells by DESN-mediated dual-model chemo-sonodynamic therapy upon low-energy ultrasound. When drugs are delivered in the body, as a result of this targeting, the drugs accumulate in tumor cells, which increases drug utilization. The dual-effect nanoparticles (DESN), with the function of accelerating the drug release at the desired site and enhancing the cavitation effect under low-energy ultrasound irradiation, have an excellent inhibiting effect on tumor cells in vivo.

**Figure 8:**
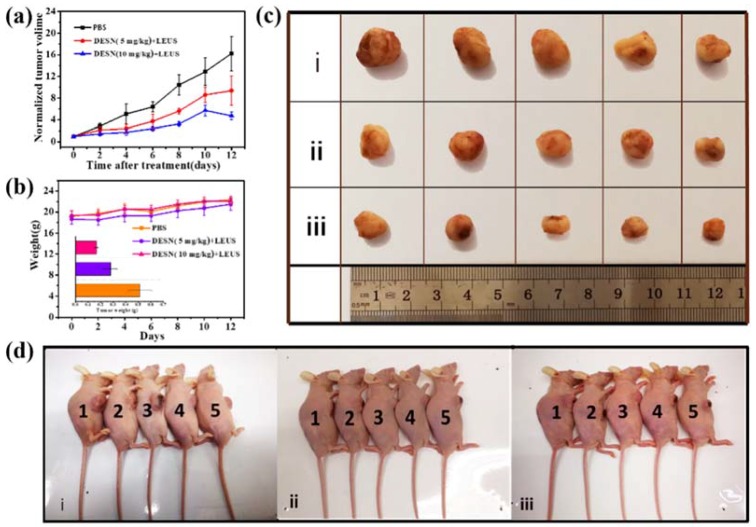
In vivo therapeutic efficacy of DESN under low-energy ultrasound irradiation: (**a**) Time-dependent tumor growth curves (*n* = 5, mean ± SD) after different treatments (control, 5 mg/kg DESN + LEUS, 10 mg/kg DESN + LEUS); (**b**) Time-dependent body-weight curves of nude mice after different treatments. Inset: tumor weights of mice 13 days after the treatments. Valued are means ± s. e. m. (*n* = 5 mice per group); (**c**) The images of tumor volume for nude mice with 4T1 cancer cells treated with blank control i, DESN (5 mg/kg) + LEUS ii, and DESN (10mg/kg) + LEUS iii and (**d**) Nude mice images of mice treated with one blank control, ii DESN (5 mg/kg) + LEUS, and iii DESN (10 mg/kg) + LEUS. The therapeutic efficacy of DESN under LEUS was obvious. As the concentration of DESN increased, the growth of the tumor was more prominent.

**Figure 9:**
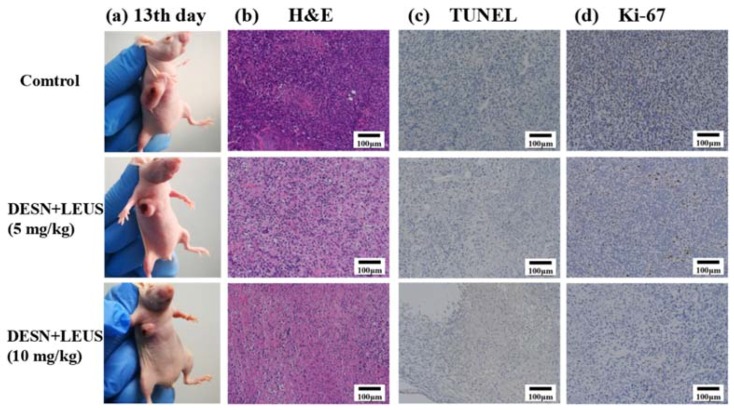
(**a**) Photographs of 4T1-tumor-bearing mice and their tumor regions 13 days after the treatment; (**b**) H&E staining for pathological changes in tumor tissues from each group to reveal the effectiveness of in vivo chemo-sonodynamic therapy by intravenous/intratumoral administration of DESN; (**c**) TUNEL staining for pathological changes in tumor tissues and (**d**) Antigen Ki-67 immunofluorescence staining for cellular proliferation in tumor sections.

Encouraged by the excellent synergistic effect in vitro and the high biocompatibility in vivo, the assay of in vivo tumor-growth inhibition experiment was further carried out on 4T1 tumor-bearing nude mice (Scheme 2). After the intratumoral administration of DESN (0 mg/kg, 5 mg/kg, and 10 mg/kg in PBS, respectively) for a certain time, the 4T1-tumor bearing mice were anesthetized and the LEUS (0.8 W·cm^−2^, 5 min) were directly irradiated on the tumor. The body weight of mice in all groups was quite stable and showed a small discrepancy, confirming that negligible adverse effects occurred during the therapeutic period after the injections (Figure 8b). Notably, the tumor-growth rates of the mice treated with DESN (10 mg/kg + LEUS) were remarkably lower than other groups treated with DESN (5 mg/kg + LEUS) or a control (PBS). Owing to the significant antitumor effect of DESN under LEUS, the tumor-growth inhibition was 45.70% for the DESN (5 mg/kg + LEUS) group, and 67.58% for DESN (10 mg/kg + LEUS) group after 13 days of treatment (Figure 8a). The inhibitory rate increased to 1.5 times as the concentration increased. The tumor sizes of the experimental groups were visibly smaller than those of the control group (Figure 8b inset, Figure 8c and Figure 9a).

The histopathological analysis of topographical tumor tissues showed pathological destruction of cell morphology and structure after the injection of DESN in H&E and TUNEL staining (Figure 9b,c). The number of apoptotic cancer cells in the experimental groups increased dramatically as compared to that of the control group, representing the effective chemo-sonodynamic therapy of DESN nanovehicles. According to antigen Ki67 assay, the proliferation of cancer cells was largely suppressed upon the administration of DESN (Figure 9d). Moreover, DESN showed diminished damage to visceral organs (heart, liver, spleen, lungs, and kidneys) compared with the control group in the histopathological examination (Figure S5). The enhanced in vivo tumor-growth inhibition was considered to originate from the efficient accumulation of DESN into tumor tissues, followed by gradual drug release from the nanocarriers accompanying the LEUS irradiation.

## 4. Conclusions

In summary, we have constructed a multifunctional mesoporous silica nano-platform for targeted chemo-sonodynamic therapy, based on mesoporous silica spheres to load hydrophobic PTX and store gas in a hydrophobic internal channel as an ultrasonic cavitation nucleus. The DESN could be targeted into cancer cells via a receptor-mediated endocytosis pathway and exhibits a high hydrophobic drug-loading capacity of up to 15.98% owing to the hydrophobic channel. Meanwhile, DESN displayed a very slow release in the bloodstream and achieved a greatly accelerated release under LEUS. As a result, the biocompatible DESN with synergistic effect of low-energy ultrasound provided the distinctively inhibiting effect on tumor cells in vitro and in vivo. The DESN (10 mg/kg) upon LEUS irradiation has a significant effect on tumor growth inhibition (V/V_0_ = 4.72 ± 0.70) in vivo, compared with the control group (V/V_0_ = 17.12 ± 2.75). This new drug delivery system is not only a LEUS synergistic agent, but also accelerates the release of the drug in the lesion site and exhibits high bio-safety, showing significant potential for future clinical applications.

## Figures and Tables

**Table 1:** Structure parameters, PTX-loading efficiency, and zeta potential of the fabricated samples (MSN, H-MSN, and DESN).

Sample	S_BET_ (m^2^/g)	D (nm)	V_pore_ (cm^3^·g^−1^)	PTX-Loading Capacity	Zeta Potential ^a^ (mV)
MSN	1000	2.38	1.089	12.4%	−16 ± 1
H-MSN	640	2.30	0.56	-	−58 ± 2
DESN	247	2.17	0.28	14.83%	65 ± 3

^a^ Zeta potential were measured in PBS (pH = 7.4) at room temperature.

## Supplementary Materials

The following are available online at http://www.mdpi.com/1996-1944/11/10/2041/s1, Figure S1: XRD patterns of MSN, H-MSN, FA-β-CD/H-MSN and DESN, Figure S2: TGA profiles of blank mesoporous silica (a); H-MSN (b); FA-β-CD/H-MSN (c) and DESN (d), Figure S3: In vivo biosafety evaluation of DESN for one month, Figure S4: Histopathological examination of the major organs of Kunming mice, Figure S5: Histopathological examination of the major organs of the 4T1 tumor bearing nude mice.
