# Supplementary material for: Mesoporous Silica Nanoparticles for Dual-Mode Chemo-Sonodynamic Therapy by Low-Energy Ultrasound
Source: Materials (Basel). 2018 Oct 19;11(10):2041. doi: 10.3390/ma11102041 (PMC6212853; doi:10.3390/ma11102041)
Supplement: Supplementary file 1 [file materials-11-02041-s001.pdf]

materials

supplementary

Mesoporous Silica Nanoparticles for Dual-Mode Chemo-Sonodynamic Therapy by Low-Energy Ultrasound

Jingjing Wang [1,2], Yajing Jiao [1] and Yiran Shao [1,*]

[1] Key Laboratory of Inorganic Coating Materials CAS, Shanghai Institute of Ceramics, Chinese Academy of Sciences, Shanghai 200050, China; 18616938063@163.com (J.W.); 18800263296@163.com (Y.J.)
[2] University of Chinese Academy of Sciences, Beijing 100049, China
* Correspondence: shaoyiran@mail.sic.ac.cn; Tel.: +86-182-2119-0389

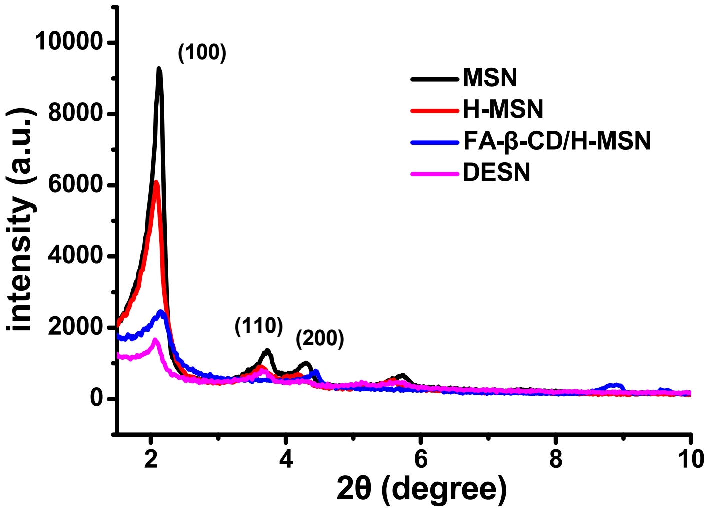

Figure S1. XRD patterns of MSN, H-MSN, FA-β-CD/H-MSN and DESN, respectively. After modification and the PTX loading, the XRD pattern of FA-β-CD/H-MSN and DESN was almost consistent with that of the MSN matrix, indicating that the meso-structure is well kept.

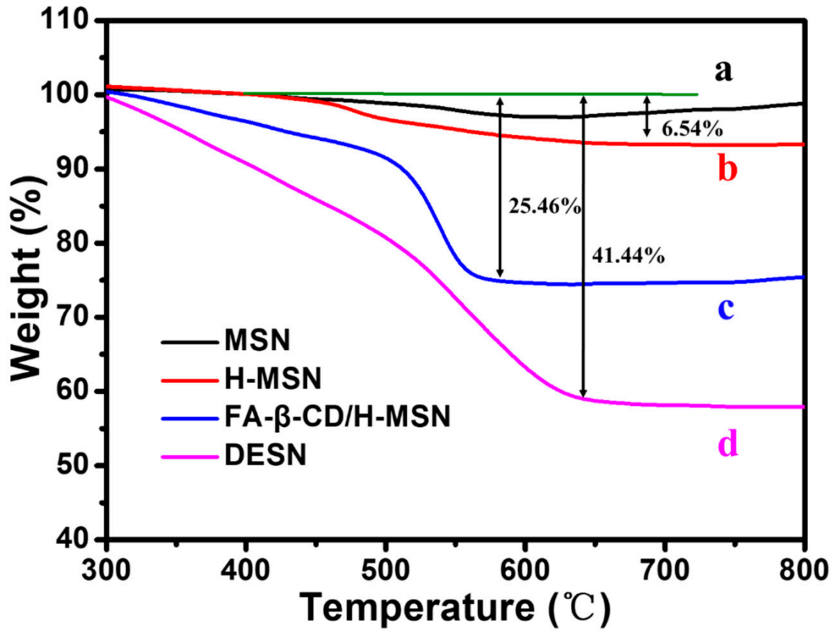

Figure S2. TGA profiles of blank mesoporous silica (**a**); H-MSN (**b**); FA-β-CD/H-MSN (**c**) and DESN (**d**).

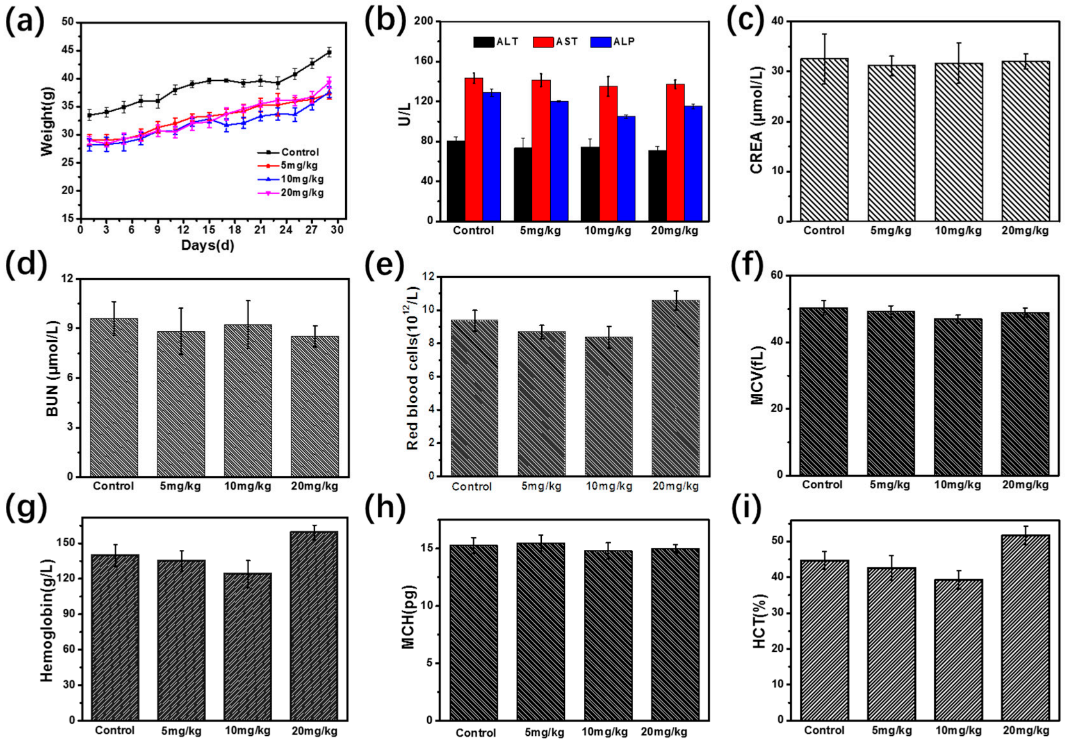

Figure S3. In vivo biosafety evaluation of DESN for one month. The Kunming mice were intravenously injected with DESN at elevated doses (5 mg/kg, 10 mg/kg and 20 mg/kg), and the pure PBS was taken as control. (**a**) Body-weight changes of experimental groups within one-month feeding duration; (**b**) Blood levels of ALT, ALP and AST as liver function markers; (**c**) Creatinine (CREA) and (**d**) blood urea nitrogen (BUN) represent as kidney function markers. The complete blood data: (**e**) Red blood cells. (**f**) mean corpuscular volume (MCV); (**g**) hemoglobin (HGB); (**h**) Mean

corpuscular hemoglobin (MCH); (**i**) Hematocrit. n = 5. Data was presented as mean ± s.d.

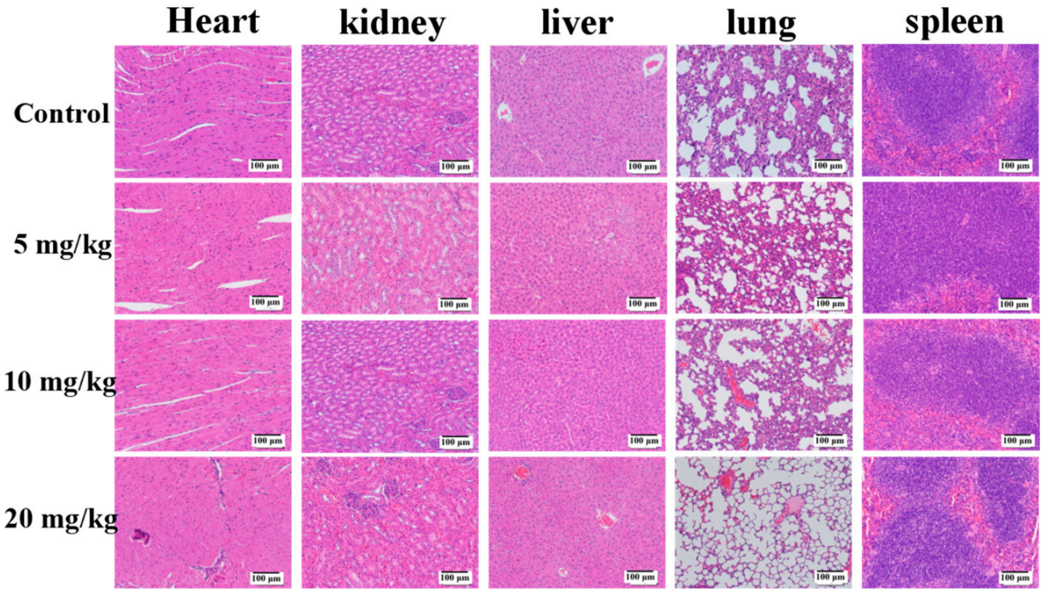

Figure S4. Histopathological examination of the major organs of Kunming mice, including heart, kidney, liver, lung and spleen. The Kunming mice were treated with DESN at elevated does (5, 10 and 20 mg/kg).

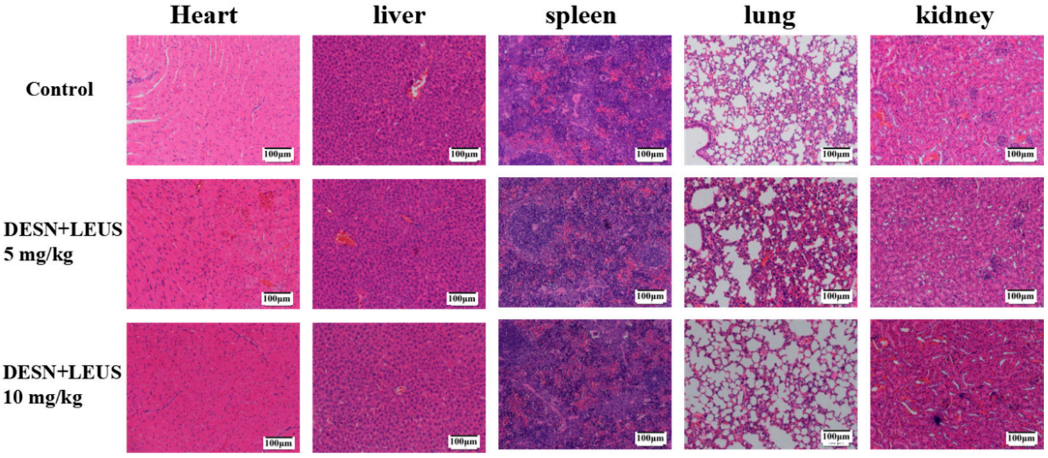

Figure S5. Histopathological examination of the major organs (heart, liver, spleen, lung and kidney) of the 4T1 tumor bearing nude mice. The mice were intravenously injected with PBS, DESN (5 mg/kg) +LEUS and DESN (10 mg/kg) +LEUS, respectively.

© 2018 by the authors. Submitted for possible open access publication under the terms and conditions of the Creative Commons Attribution (CC BY) license (http://creativecommons.org/licenses/by/4.0/).
